# Supplementary figures and images for: Hi-MC: a novel method for high-throughput mitochondrial haplogroup classification
Source: PeerJ. 2018 Jun 25;6:e5149. doi: 10.7717/peerj.5149 (PMC6022720; doi:10.7717/peerj.5149)

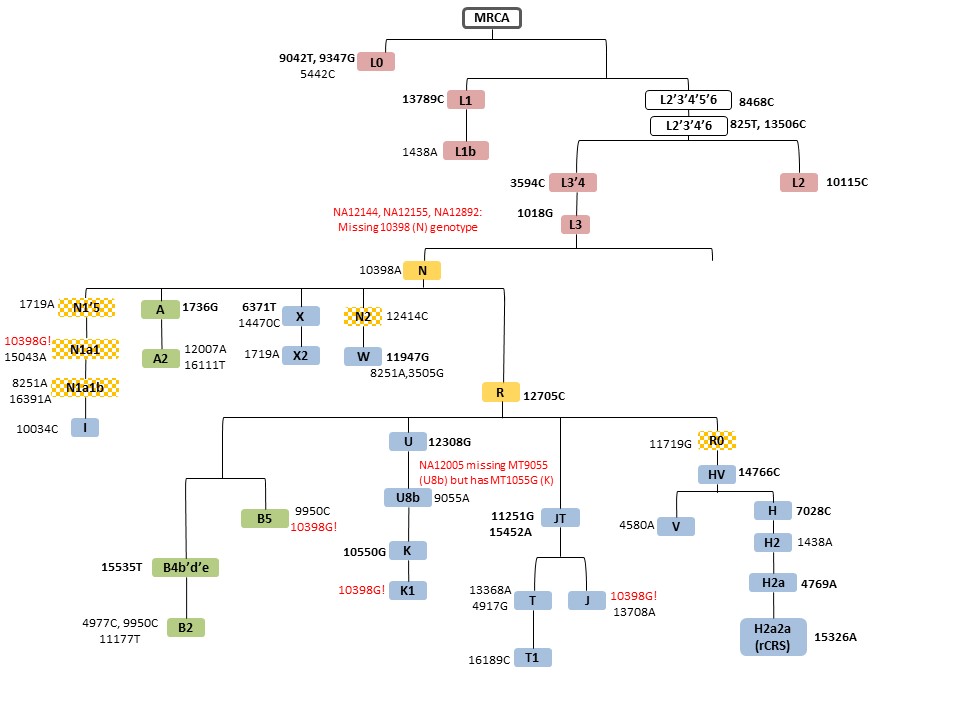

Supplement: Supplemental Information 2 — Select haplogroup misclassifications are mapped to the simplified mitochondrial phylogenetic tree from Mitchell et al 2014 (PMID:24488180) for CEU (Fig. S1), YRI (Fig. S2), and CHB/JPT (Fig. S3). For each Supplementary Figure, mitochondrial SNPs and tree branches are color coded by population: European (blue), African (red), and Native American/Asian (green) mitochondrial haplogroup lineages. Eurasian macrohaplogroups are in orange and the checkered boxes indicate haplogroups that were included in counts of the corresponding parent Eurasian macrohaplogroup. mtSNPs in bold are found in a single haplogroup and those followed by (!) indicate reversions. [file peerj-06-5149-s002.jpg]

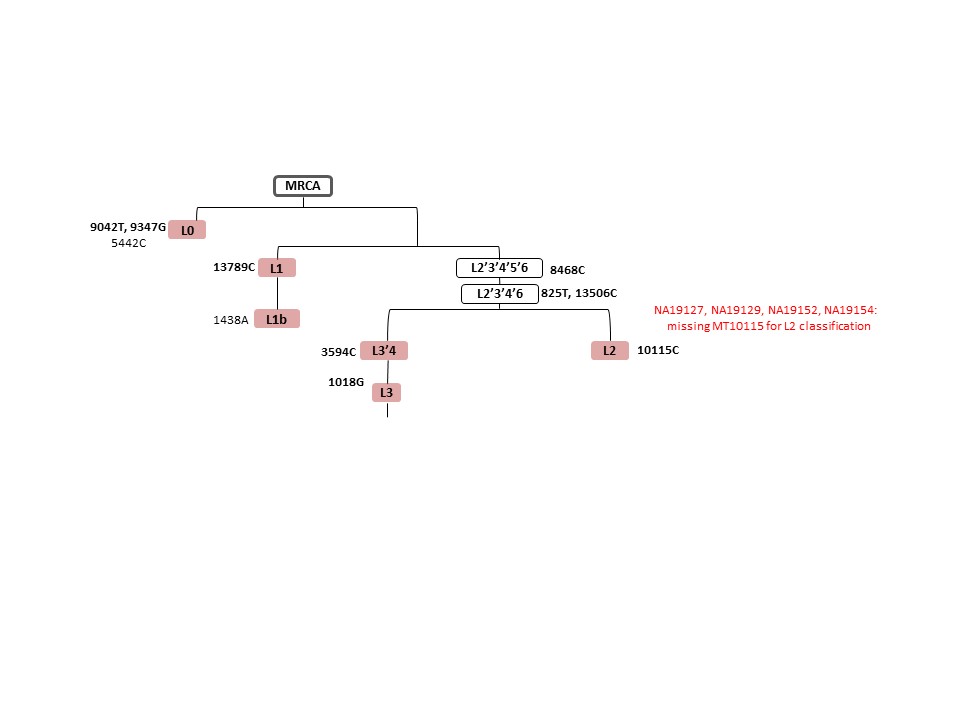

Supplement: Supplemental Information 3 — Select haplogroup misclassifications are mapped to the simplified mitochondrial phylogenetic tree from Mitchell et al 2014 (PMID:24488180) for CEU (Fig. S1), YRI (Fig. S2), and CHB/JPT (Fig. S3). For each Supplementary Figure, mitochondrial SNPs and tree branches are color coded by population: European (blue), African (red), and Native American/Asian (green) mitochondrial haplogroup lineages. Eurasian macrohaplogroups are in orange and the checkered boxes indicate haplogroups that were included in counts of the corresponding parent Eurasian macrohaplogroup. mtSNPs in bold are found in a single haplogroup and those followed by (!) indicate reversions. [file peerj-06-5149-s003.jpg]

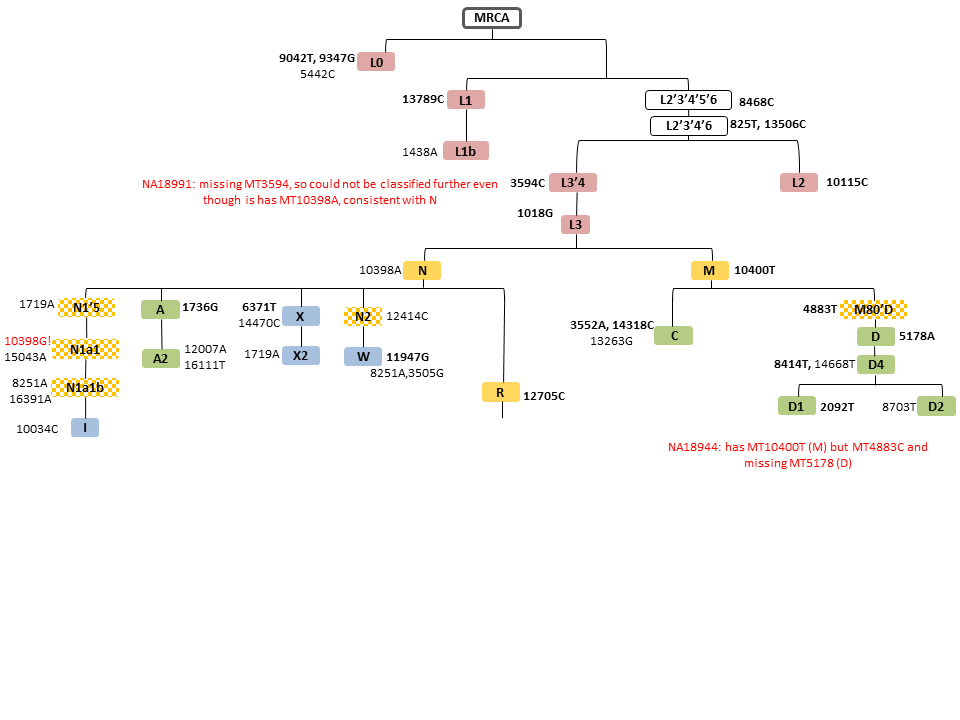

Supplement: Supplemental Information 4 — Select haplogroup misclassifications are mapped to the simplified mitochondrial phylogenetic tree from Mitchell et al 2014 (PMID:24488180) for CEU (Fig. S1), YRI (Fig. S2), and CHB/JPT (Fig. S3). For each Supplementary Figure, mitochondrial SNPs and tree branches are color coded by population: European (blue), African (red), and Native American/Asian (green) mitochondrial haplogroup lineages. Eurasian macrohaplogroups are in orange and the checkered boxes indicate haplogroups that were included in counts of the corresponding parent Eurasian macrohaplogroup. mtSNPs in bold are found in a single haplogroup and those followed by (!) indicate reversions. [file peerj-06-5149-s004.png]
